# Editorial: Advancing Health Services for Vulnerable Populations Including Those with Chronic Conditions

**DOI:** 10.3390/ijerph23030312

**Published:** 2026-03-02

**Authors:** Chiung-Jung Wu, Xiang-Yu Hou, Lijun Fan

**Affiliations:** 1 Center of Innovation in Health Sciences, College of Health Sciences, VinUniversity, Hanoi 100000, Vietnam; 2 School of Health, University of the Sunshine Coast, Petrie, QLD 4502, Australia; 3 Royal Brisbane and Women’s Hospital (RBWH), Herston, QLD 4006, Australia; 4 Shinawatra University, Pathum Thani 12160, Thailand; 5 Broken Hill University Department of Rural Health, Susan Wakil School of Nursing and Midwifery, Faculty of Medicine and Health, The University of Sydney, Sydney, NSW 2880, Australia; xiang-yu.hou@sydney.edu.au; 6 School of Public Health, Southeast University, Nanjing 210009, China; fanlijun@seu.edu.cn

Vulnerable populations include low-income individuals and families, ethnic minorities living in rural areas, older adults, and people with disabilities [1]. The population at risk for chronic diseases may experience daily social, physical, economic, and environmental challenges [2]. Advancing health services for vulnerable populations by reducing avoidable disparities and ensuring equitable access to high-quality, culturally responsive care is paramount [3].

In this Special Issue, *Advancing Health Services for Vulnerable Populations, including those with chronic conditions*, we bring together 11 papers that address key challenges in healthcare delivery. As outlined in the call for papers, these contributions examine risk factors, self-management, and health service delivery for populations who frequently fall through gaps in healthcare systems. The studies are geographically diverse, spanning Australia, the United States, Switzerland, and Sudan, and methodologically broad, ranging from big-data analyses to qualitative interviews. Collectively, they deliver a coherent message: improving outcomes for vulnerable populations requires sustained attention to comorbidities, barriers to access, and context-specific interventions.

*At-Risk Population Tends to Have Poorer Health Outcomes*

This at-risk population not only experiences a burden of multiple co-existing comorbidities but also experiences disproportionately poorer health outcomes, including a higher rate of disease progression, increased morbidity and mortality, and greater reliance on healthcare services [4]. Evidence from Ben Zvi et al.’s **(contribution 1)** analysis of a U.S. hospital database demonstrates that patients with diabetic foot ulcers (DFU) who also have comorbidities, such as sepsis, stroke, or kidney disease, have a significantly increased likelihood of major amputation and in-hospital mortality. Rodwell’s Australian survey data similarly show that health problems worsened physical function and reduced work ability, all of which predicted future hospital admission among adults living alone **(contribution 2)**.

*Challenges for Healthcare Systems*

With aging populations and their growing impact on healthcare systems, vulnerable groups are increasingly affected. Many countries face escalating health and social challenges, including chronic diseases. These trends place substantial strain on healthcare systems through rising costs, increased demand for services, and workforce shortages [5,6].

The challenges are further compounded by global inequities. For example, Gebril et al. **(contribution 3)** highlight the need for future investigations into local risk factors (e.g., tobacco, viruses) in head-and-neck tumors, as well as the importance of building capacity through the establishment of a national cancer registry in Sudan **(contribution 3)**. Such infrastructure, including registries and a routine data collection system, is as crucial as the interventions themselves. This global perspective complements findings from the Australian and North American studies by underscoring that *health systems strengthening* (spanning data infrastructure, workforce capacity, and service delivery) remains a fundamental requirement, particularly in resource-constrained and vulnerable settings.

*Complex Needs Require Innovative Interventions*

Patients living with multiple comorbid conditions often present with complex and interrelated healthcare needs, necessitating coordinated, integrated, and person-centered models of care to ensure effective management, improve health outcomes, and optimize the use of healthcare resources [7]. The care needs to be focused on the patient’s voice and experience. For example, Gulliver et al.’s study reported that cancer survivors voiced “*difficulties around access to and management of services for both their mental and physical health*” (**contribution 4**). Similarly, Sanjida et al. reported that health professionals expressed their concerns about *communication* (language, health literacy, and understanding of cancer pathways), *cultural safety* (trust, privacy, and racism), and *service access* (especially during transitions between care levels) **(contribution 5)**.

Our published papers highlight that there is a gap between optimal care and real-world practice. For example, Watt et al.’s clinical audit in Queensland hospitals in Australia revealed a striking mismatch that although 90% of elderly inpatients who fell met the criteria for a CT head scan, only half actually received one (**contribution 6**). Liddle et al. reported that remote Aboriginal communities value the holistic, culturally responsive care offered by their community clinics, but worry about high staff turnover (**contribution 7**). Notably, community members showed pragmatism by accepting rotated or job-sharing nurses and doctors if this helped build trust and continuity. These findings jointly suggest that policies and programs must be tailored to local contexts and realities.

*Diverse Methods and Future Research*

Methodologically, this Special Issue is impressively diverse. We see large-scale quantitative studies **(contribution 1, contribution 2)**, longitudinal cohorts studies **(contribution 8)**, registry analytics (Sudan’s National Cancer Institute data), and mixed-methods projects **(contribution 9, contribution 7)**, as well as a narrative review **(contribution 5)**. However, longitudinal tracking (beyond Rodwell’s work) **(contribution 2)** and experimental trials are relatively rare. For instance, we still need prospective studies to test whether the interventions suggested by stakeholders (better food access, telehealth, workforce incentives) actually improve health outcomes. Editorially, we note that future research could build on these mixed methods. For example, this could include piloting an integrated primary-care model in an Aboriginal community clinic or formally evaluating a produce voucher program with control groups. In sum, combining big data analytics with on-the-ground engagement offers a holistic view but also invites the next step of ‘what works’.

Looking ahead, these published papers point to clear priorities. First, we must translate insights into policy and practice. For example, the finding that DFU patients with sepsis or heart attack have the worst prognosis suggests prioritizing them for multidisciplinary foot clinics or early surgery. The CT-scan audit suggests revising the Post-Fall pathway or training to ensure no high-risk older patient is missed **(contribution 6)**. Second, interventions should be co-designed with communities. Stakeholder and user feedback indicates that top-down solutions will falter if they ignore cultural or contextual nuances. Third, we need to address social determinants alongside medical care. Chronic disease care cannot be siloed from issues like poverty and the environment. Fourth, capacity building in low-resource settings is essential. Finally, each paper highlights questions for future study. How can we scale up allied-health access for isolated patients? Will expanding the Indigenous health workforce measurably improve cancer outcomes? Could routine functionality checks avert hospitalizations in lonely seniors? These questions, and more, suggest a rich agenda.

This Special Issue demonstrates both progress and remaining work. Progress is evident in the new data and innovative interventions. Yet the “action gap” is also clear, as vulnerabilities persist when health systems are under-resourced or fragmented. We hope these papers inspire continued interdisciplinary effort, bringing together clinicians, public health specialists, community leaders, and researchers, to turn evidence into equity. As editors, we thank the authors for their contributions and look forward to seeing how the field builds on this foundation to better serve those most in need of care.
